# Access to influenza immunisation services by HIV‐positive patients in the UK

**DOI:** 10.1111/irv.12473

**Published:** 2018-03-30

**Authors:** Elisha Pickett, James Brown, May van Schalkwyk, Alan Hunter, Kelly Edwards, Sarah Edwards, Neal Marshall, Leonie Swaden, Fiona Burns, Margaret Johnson, Marc Lipman

**Affiliations:** ^1^ Departments of HIV and Respiratory Medicine Royal Free London NHS Foundation Trust London UK; ^2^ UCL Respiratory Division of Medicine University College London London UK; ^3^ Research Dept. of Infection & Population Health University College London London UK

Influenza is an important cause of morbidity in HIV‐positive adults, who may be more susceptible and more likely to develop severe disease.^1^, ^2^ Annual influenza immunisation is recommended for all HIV‐positive adults in the UK, supported by British HIV Association (BHIVA) guidelines,^1^ with evidence for reasonably good uptake.^3^ HIV services do not receive specific funding to provide immunisation; and the National Flu Immunisation Programme offers this instead via primary care and pharmacies.^4^ Whether this meets the needs of people living with HIV has not been evaluated.

To inform the design of services for influenza immunisation we undertook a survey of adults attending a metropolitan HIV service during autumn/winter 2015‐2016. Participants were asked about their behaviour regarding influenza immunisation and preferences for services used to obtain this (Appendix). We obtained written consent to access clinical records and contact participants later in the season to establish whether immunisation had been received. We also documented whether participants consented to share details of their HIV status with their GP (General Practitioner), as we hypothesised that this might affect their ability to access influenza immunisation.

A total of 253 individuals participated: their median age was 48 years (IQR 41‐54); 80% were male; 62% were White, 22% were Black and 4% were of Asian ethnicity. The median blood CD4 count was 627 cells/μL (IQR 434‐873), 96% were using antiretroviral therapy, and 76% had an undetectable HIV load.

Overall, 212 participants (84%) were aware of the recommendation for annual influenza immunisation. A total of 176 individuals (70%) were immunised during the 2015‐2016 influenza season, 53 (21%) were not and in 24 (9%) we had no record of immunisation status as they did not respond to follow‐up. Uptake did not differ significantly by gender, ethnicity, CD4 count or HIV load; those immunised were slightly older than those who were not (median 49 compared to 46 years, *P* = .005) (Table 1).

**Table 1:** Relationship between participant characteristics and uptake of immunisation

	Was participant immunised in 2015‐2016 season?			*P* value
	Yes (n = 176)	No (n = 53)	Unknown (n = 24)	
Gender^c^				
Female n (row %)	37 (68)	12 (23)	5 (9)	.92
Male n (row %)	136 (71)	38 (20)	18 (9)	
Race/ethnic origin				
White n (row %)	109 (70)	34 (22)	13 (8)	.884
Black n (row %)	35 (64)	12 (22)	8 (14.5)	
Other n (row %)	16 (80)	3 (15)	1 (5)	
Mixed n (row %)	6 (86)	1 (14)	0	
Not stated n (row %)	10 (67)	3 (20)	2 (13)	
Age, years, median (IQR)	49 (43‐55)	46 (39‐50)	44 (39‐51)	.005^b^
Blood CD4 count (cells/µL) median (IQR)^d^	625 (429‐867)	627 (473‐881)	636 (459‐840)	.979^b^
Plasma HIV load <40 copies/mL^d^ n (row %)				
Yes	143 (71.5)	40 (20)	17 (8)	.413^a^
No	25 (66)	9 (24)	4 (10)	
Consent to contact General practitioner (GP) n (row %)				
Yes	134 (74)	35 (20)	11 (6)	.05^e^
No	30 (61)	11 (22)	8 (16)	

a Chi‐Squared test.

b Kruskal‐Wallis test.

c 3 participants did not state their gender.

d blood results available for 240 participants.

e calculation excludes 25 participants with no details regarding consent to GP contact.

Of those immunised, 90 (51%) had this in their GP practice, 44 (25%) in an HIV service, 15 (9%) in a pharmacy and 20 (11%) elsewhere (7 individuals did not specify a location). Those immunised in their GP practice or pharmacy were older than those immunised in an HIV care service (mean age 50 and 49 years for those immunised in GP or pharmacies, respectively and 45 years for those immunised in HIV care services, *P* = .004). Consent to share information between HIV services and primary care appeared to influence uptake: overall, 180 participants (71%) consented to share details of their HIV status with their GP and 49 (19%) did not (for 24 participants this was not recorded). Uptake of influenza immunisation was higher in those consenting to data sharing (74% vs 61%, *P* = .05); and individuals immunised in primary care were more likely to consent to this than those immunised by HIV services (83% vs 67%, *P* = .013).

Our data suggest that HIV‐positive adults have a reasonable uptake of influenza immunisation and around half of these immunisations are provided in primary care. Uptake was higher amongst people who consented to share details of their HIV status with their GP, and 25% of immunisations were provided by the HIV service. This suggests that the current (reasonably good) uptake of influenza immunisation relies on significant provision by HIV care providers.

The 2015 BHIVA guidelines on the immunisation of HIV‐positive adults suggest that HIV services should work in partnership with primary care to ensure that patients receive annual immunisation.^1^ Exploring the reasons why individuals do not want to share information between HIV services and primary care, and creating mechanisms for sharing clinical data (particularly in a way that is accessible to service users) could enable better targeting of immunisations for those who do not receive these in primary care.

## Promoting Influenza Immunisation In People Attending the ICDC Winter 2015‐16

This questionnaire is about your views on having the influenza immunisation. We will use the information obtained to try and improve services both here and at other sites. You do not have to complete this questionnaire, and if you do not want to answer any questions just leave them blank.

Please note that all information provided will be used for research purposes and to improve our patient services. The data will only be accessed by professional staff and will be stored securely in accordance with hospital data protection policy.

Please complete the following questions, marking yes or no as appropriate and providing further details if relevant in the space provided.
